# Supplementary material for: Fabrication of Eco-Friendly Betanin Hybrid Materials Based on Palygorskite and Halloysite
Source: Materials (Basel). 2020 Oct 18;13(20):4649. doi: 10.3390/ma13204649 (PMC7603274; doi:10.3390/ma13204649)
Supplement: Supplementary file 1 [file materials-13-04649-s001.pdf]

materials

MDPI

Supplementary Materials

Fabrication of Eco-Friendly Betanin Hybrid Materials Based on Palygorskite and Halloysite

Shue Li [1,2,3], Bin Mu [1,3,*], Xiaowen Wang [1,3], Yuru Kang [1,3] and Aiqin Wang [1,3,*]

1 Key Laboratory of Clay Mineral Applied Research of Gansu Province, Center of Eco-Materials and Green Chemistry, Lanzhou Institute of Chemical Physics, Chinese Academy of Sciences, Lanzhou 730000, China; seli17@licp.cas.cn (S.L.); wangxw@licp.cas.cn (X.W.); yurukang@licp.cas.cn (Y.K.)

2 Center of Materials Science and Optoelectronics Engineering, University of Chinese Academy of Sciences, Beijing 100049, China

3 Center of Xuyi Palygorskite Applied Technology, Lanzhou Institute of Chemical Physics, Chinese Academy of Sciences, Xuyi 211700, China

* Correspondence: mubin@licp.cas.cn (B.M.); aqwang@licp.cas.cn (A.W.); Fax: +86-931-496-8019; Tel: +86-931-486-8118

Received: 3 September 2020; Accepted: 15 October 2020; Published: date

I. Supplementary Figures

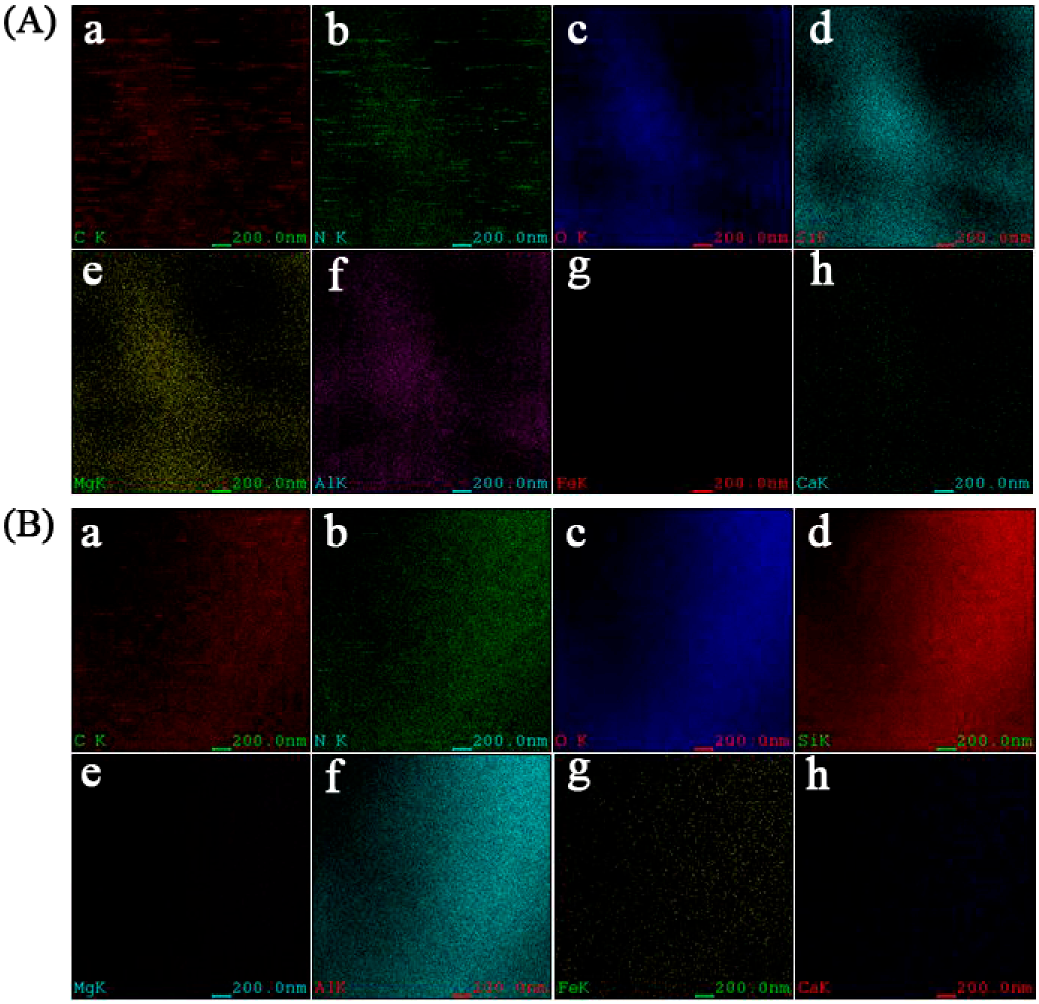

Figure S1. The element mapping images of (**A**) betanin/Pal and (**B**) betanin/Hal: (**a**) C, (**b**) N, (**c**) O, (**d**) Si, (**e**) Mg, (**f**), Al (**g**) Fe, (**h**) Ca.

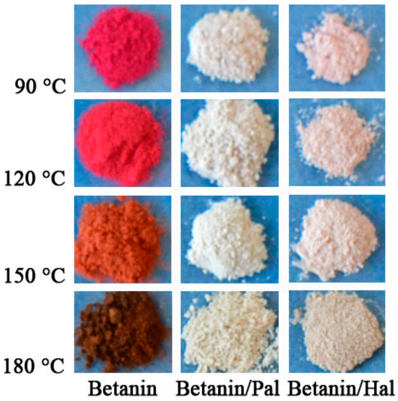

Figure S2. Digital images of the pure betanin, betanin/Pal and betanin/Hal at different heating temperatures.

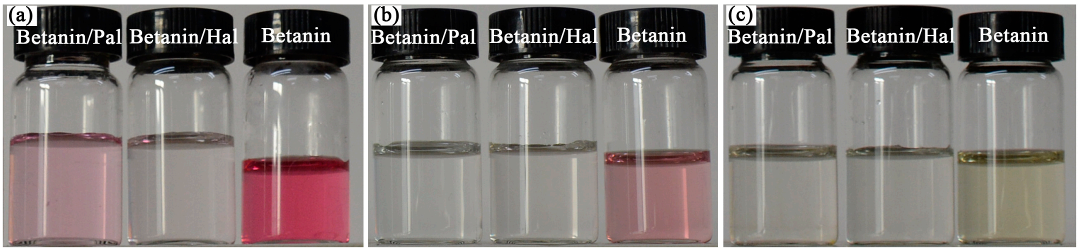

Figure S3. Digital images of the supernate after the pure betanin, betanin/Pal, betanin/Hal were immersed h in (**a**) distilled water, (**b**) 0.1 M HCl and (**c**) 0.1 M NaOH for 24, respectively.

II. Supplementary Tables

Table S1. Color parameters of pure betanin, Pal and Hal.

Samples	Color Parameters		
	L^*	a^*	b^*
**Betanin**	64.54	15.13	5.99
**Pal**	83.08	0.41	6.27
**Hal**	77.10	4.86	11.95

Table 2. Chemical compositions and their atomic percents of Pal, betanin/Pal, Hal, and betanin/Hal.

Elements	Atomic Percents (%)			
	Pal	Betanin/Pal	Hal	Betanin/Hal
C	37.06	58.37	52.87	50.33
N	1.51	1.43	0.98	0.79
O	35.03	22.79	26.03	27.23
Si	17.38	10.49	10.93	11.71
Mg	1.51	1.04	0.46	0.35
Al	4.00	3.28	6.95	7.39
Fe	0.76	0.60	0.48	0.42
Ca	1.77	1.34	0.46	0.4
Na	0.52	0.32	0.38	0.45
K	0.47	0.34	0.46	0.93

© 2020 by the authors. Submitted for possible open access publication under the terms and conditions of the Creative Commons Attribution (CC BY) license (http://creativecommons.org/licenses/by/4.0/).
